# The vanishing touch: Unveiling the tuft erosion in scleroderma

**DOI:** 10.1515/rir-2024-0008

**Published:** 2024-03-31

**Authors:** Hui Jiang, Yangzhong Zhou

**Affiliations:** Department of Rheumatology and Clinical Immunology, Chinese Academy of Medical Sciences & Peking Union Medical College; Beijing, China; National Clinical Research Center for Dermatologic and Immunologic Diseases, Ministry of Science & Technology; Beijing, China; State Key Laboratory of Complex Severe and Rare Diseases, Peking Union Medical College Hospital; Beijing, China; Key Laboratory of Rheumatology and Clinical Immunology, Ministry of Education; Beijing, China

**Figure 1:**
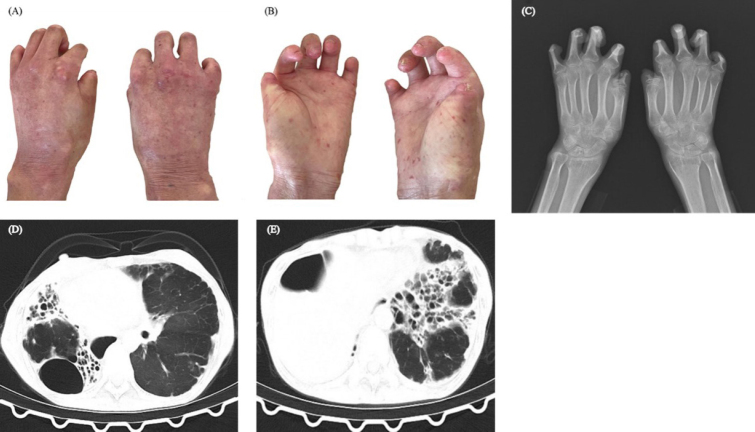
*A, B: Flexion contractures of the interphalangeal joints. C: Hands X-ray presented acro-osteolysis. D, E: CT scan presented severe interstitial lung disease*.

A 52-year-old woman presented to our clinic complaining of stiffness and shortened fingers. She had a 30-year history of swollen fingers, sclerodactyly, fingertip ulcers, telangiectasias, and subcutaneous calcification. In the last three years, she developed dyspnea, exercise intolerance, and gastroesophageal reflux. Physical examination revealed flexion contractures of the interphalangeal joints, and the distal part of her fingers disappeared. (Figure 1A, B). Laboratory tests showed positive anticentromere antibody. Hands X-ray revealed acro-osteolysis presenting a picture like a “pencil-incup” ^[1]^ Bilateral wrist X-ray showed mild joint space narrowing, while the metacarpophalangeal joints appeared unaffected (Figure 1C). Chest computer tomography (CT) disclosed severe interstitial lung disease and extensive destruction across both lungs (Figure 1D, E). The patient was treated with prednisone 30 mg daily, combined with mycophenolate mofetil 0.5 g twice daily, tadalafil 10 mg daily, and pirfenidone.

Acro-osteolysis, a typical complication of scleroderma, is caused by chronic ischemic changes of the digital tuft due to an extended period of Raynaud’s syndrome.^[2,3]^ Acroosteolysis may lead to diminished tactile sensitivity and undermine the structural integrity of fingers, metaphorically described as “The Vanishing Touch”. Early recognition and treatment may potentially prevent extensive bone tissue destruction.
